# Identification of spatial patterns with maximum association between power of resting state neural oscillations and trait anxiety

**DOI:** 10.1007/s00521-022-07847-5

**Published:** 2022-10-01

**Authors:** Carmen Vidaurre, Vadim V. Nikulin, Maria Herrojo Ruiz

**Affiliations:** 1 Neuroengineering Group, TECNALIA, Basque Research and Technology Alliance (BRTA), Donostia-San Sebastian, Spain; 2 IKERBASQUE, Basque Foundation for Science, Bilbao, Spain; 3 Department of Statistics, Computer Science and Mathematics, Public University of Navarre, Pamplona, Spain; 4 Department of Neurology, Max Planck Institute for Human Cognitive and Brain Sciences, Leipzig, Germany; 5 Institute for Cognitive Neuroscience, National Research University Higher School of Economics, Moscow, Russian Federation; 6 Psychology Department, Goldsmiths University of London, London, UK

**Keywords:** EEG/MEG oscillations, Anxiety, Supervised spatial patterns, Affective neurofeedback, Affective interface, Emotion neurofeedback

## Abstract

Anxiety affects approximately 5–10% of the adult population worldwide, placing a large burden on the health systems. Despite its omnipresence and impact on mental and physical health, most of the individuals affected by anxiety do not receive appropriate treatment. Current research in the field of psychiatry emphasizes the need to identify and validate biological markers relevant to this condition. Neurophysiological preclinical studies are a prominent approach to determine brain rhythms that can be reliable markers of key features of anxiety. However, while neuroimaging research consistently implicated prefrontal cortex and subcortical structures, such as amygdala and hippocampus, in anxiety, there is still a lack of consensus on the underlying neurophysiological processes contributing to this condition. Methods allowing non-invasive recording and assessment of cortical processing may provide an opportunity to help identify anxiety signatures that could be used as intervention targets. In this study, we apply Source-Power Comodulation (SPoC) to electroencephalography (EEG) recordings in a sample of participants with different levels of trait anxiety. SPoC was developed to find spatial filters and patterns whose power comodulates with an external variable in individual participants. The obtained patterns can be interpreted neurophysiologically. Here, we extend the use of SPoC to a multi-subject setting and test its validity using simulated data with a realistic head model. Next, we apply our SPoC framework to resting state EEG of 43 human participants for whom trait anxiety scores were available. SPoC inter-subject analysis of narrow frequency band data reveals neurophysiologically meaningful spatial patterns in the theta band (4–7 Hz) that are negatively correlated with anxiety. The outcome is specific to the theta band and not observed in the alpha (8–12 Hz) or beta (13–30 Hz) frequency range. The theta-band spatial pattern is primarily localised to the superior frontal gyrus. We discuss the relevance of our spatial pattern results for the search of biomarkers for anxiety and their application in neurofeedback studies.

## Introduction

Anxiety disorders are one of the leading causes of the global health-related burden. The COVID-19 pandemic had a significant impact on the prevalence of anxiety disorders worldwide, leading to an estimated increase of 25.6 % globally (374 million in total [1]). This calls for a need to identify novel therapeutic approaches that can complement established pharmacological treatment protocols. The use of non-invasive techniques to record brain activity with high temporal resolution, such as Electroencephalography (EEG) or Magnetoencephalography (MEG), provides an opportunity to assess changes in the dynamics of neural activity associated with anxiety. The analysis of neural oscillations, in particular, is ideally suited to identify markers of aberrant physiological processing [2] in neuropsychiatric conditions. By linking alterations in neural oscillations to clinical and subclinical manifestations of anxiety, it is possible to define novel neurophysiological targets for neuromodulatory and neurofeedback interventions [3], as well as for pharmacological treatment [4].

In recent years, an enormous effort has been devoted to understanding the neurobiology of anxiety disorders, combining animal work with human neuroimaging studies in healthy and clinical populations. Converging neuroimaging evidence in clinical and subclinical anxiety indicates that alterations in dorsal medial prefrontal (anterior cingulate) cortex and subcortical brain regions can explain an array of cognitive-affective alterations in these populations [5–7]. On a neurophysiological level, EEG recordings in clinical and subclinical populations have identified cross-frequency correlations as a candidate marker for anxiety disorders [8–12]. In particular, alterations in the amplitude-amplitude cross-frequency correlations (AAC) between delta ($< 4$ Hz) and beta (13–30 Hz) oscillations have been associated with social anxiety and with aberrant stress regulatory processes [9, 10]. The direction of the effect remained, however, unclear, as increased delta-beta AAC at frontal regions was associated both with a pronounced increase in social anxiety [8] and reduced trait anxiety [13]. Analysis of phase-amplitude coupling (PAC), which is a different measure of information transfer between neuronal populations, has provided more consistent results, exhibiting a reliable modulation on a within-subject level following a social anxiety manipulation [11]. Despite suggestions that delta-beta AAC and PAC could reflect altered coupling between frontal and sub-cortical circuits implicated in anxiety disorders, the functional significance of these effects remains elusive.

An alternative EEG marker of aberrant neural dynamics in anxiety conditions could be the oscillatory power over frontal regions. Measures of alpha power (8–12 Hz) have been used to obtain the index of frontal alpha asymmetry (FAA), which is sensitive to a range of emotional changes including anxiety [14, 15]. Attenuated or enhanced right relative to left frontal alpha power has been associated with approach or withdrawal motivation, respectively [16, 17]. Given this association, FAA can inform about an array of clinical mood and anxiety disorders that interfere with approach-avoidance behaviour, such as major depression, bipolar disorder, panic and anxiety disorder [18, 19]. However, the direction of the association between FAA and affective state, as well as the sign of change in alpha power are often inconsistent [15, 20, 21]. Moreover, in cases of comorbid anxiety and depression, it is unclear how reliable the FAA index can be. These inconsistencies pose a challenge for mental health research acknowledged in previous work, as a reliable and unique association between FAA and specific psychiatric conditions seems elusive [22, 23].

Animal studies suggest hippocampal theta oscillations (4–7 Hz; extended to 4–12 Hz in the rodent literature) as an important marker of anxiety, due to their involvement in the modulation of the behavioural inhibition system that is associated with processing approach-avoidance conflict [24, 25]. Human studies validate the role of theta oscillations in anxiety, with novel data demonstrating in clinical and subclinical samples that anxiolytic drugs reduce frontal theta (and alpha) oscillations during conflict processing [4]. Consistent with those findings, earlier work demonstrated heightened frontal theta power in healthy individuals with high trait anxiety levels, which was accompanied by overly cautious and avoidant behaviour [26]. Theta power at rest, by contrast, is negatively correlated with anxiety, as shown in individuals with social phobia [27].

Although resting-state studies in anxiety conditions are scarce, the published findings have been used to design neurofeedback protocols. Neurofeedback training is a non-pharmacological and non-invasive neuromodulatory approach to modify neural activity in real time using brain-computer interfaces (BCI, [2, 28–34]). Theta and alpha rhythms are the preferred signals in EEG-based neurofeedback studies aiming to mitigate anxiety and arousal, as well as promote relaxation (see recent review: [3]). The evidence so far is promising but inconclusive. Reasons are limited sample sizes, suboptimal study design and lack of control of confounding factors, such as the closed-loop interactions between learning and anxiety, which could negatively influence how participants learn from neurofeedback [3]. We suggest that an additional issue in previous neurofeedback interventions is the lack of consensus on the spatial distribution of the EEG oscillatory modulations targeted. Indeed, previous correlation analyses between oscillations and clinical or subclinical anxiety features were conducted in the sensor space, which may lead to inconsistencies due to the mixture of noise and source signals. Accordingly, sensor-based scalp distributions of oscillatory measures may not be optimal as targets for neurofeedback and BCI. Moreover, the spatial distribution of these sensor-based correlation results cannot be interpreted in terms of source activity and, thus, is less informative. A potential answer to these inconsistencies lies in methods that provide not only optimal solutions for the search of associations between neuronal measures and behavioural or psychological parameters, but also neurophysiologically interpretable results.

In this work we present results of a novel framework to extract invariant sources correlated to trait anxiety across subjects. It consists of a dimensionality reduction coupled with a spatial filtering technique called Source Power Comodulation (SPoC, [35]). SPoC was designed to find filters and patterns of sources whose power maximally comodulates with an external behavioural or psychological variable. By construction, the obtained sources are optimally related to the external variable, and can thus easily be used as neurofeedback target in online paradigms. Previously, SPoC was validated using simulated and experimental data of single participants [35]. In that study, the EEG activity of each participant was assessed to identify modulation by an external variable across trials (reaction times). In the current study, we implement SPoC on a multi-subject level whereby each participant is represented as one trial, and the value of the external variable describes a feature of each individual: trait anxiety. First, we perform the analysis on simulated data with realistic head models to assess whether SPoC is robust under different noise levels. Next, we apply SPoC to EEG resting data of 43 participants with varying levels of trait anxiety. This approach allows us to identify patterns of sources whose power at specific frequency bands maximally comodulates with trait anxiety scores.

Multi-subject SPoC allows finding patterns more strongly correlated to anxiety than usual sensor-based correlation analyses. In contrast to sensor-space correlations, SPoC results can be directly transformed to source activity using standard approaches, such as eLORETA [36]. Accordingly, SPoC results can be interpreted in terms of neurophysiological sources. The identified pattern could thus be used to inform future neuromodulation studies (e.g. non-invasive brain stimulation, neurofeedback) aiming to mitigate features associated with anxiety.

## Data description

### Simulated EEG data

SPoC was originally developed to find subject-specific patterns from trial-wise data of a single participant. In the current study we extend the use of SPoC to several participants and assess its ability to find patterns of comodulation between participants’ neural and behavioural or psychological variables. This use of SPoC can be implemented by defining each of the epochs as the data of one participant. To the best of our knowledge, this approach was previously tested only in our previous work [37]. In the present paper, we first validated it by conducting SPoC on simulated data using a realistic head model, where SSD was previously applied to reduce dimensions and reduce the risk of over fit.

Simulated data were generated by fitting 30 EEG channels to the outermost layer of the standard Montreal Neurological Institute (MNI) head [38]. The EEG forward solutions were obtained with a head model based on a three compartment realistic volume conductor [39].

The brain sources were modelled as pseudo-random cortical dipoles, defined by three location and three orientation variables. For each subject five brain sources were generated. One of them had only approximately the same location in all participants. This was done to individually simulate the different brain folding of each subject. It was achieved by including random orientation variability of up to $\pm 10\%$ in the “invariant” source. The other sources had different random positions for each participant (in each trial). Background EEG noise was also generated with 500 uncorrelated dipoles of random orientation and distribution on the cortex with 1/*f* type spectrum. These dipoles differed from person to person (trial to trial) to simulate the different background noise of each participant.

EEG oscillations were generated by band-pass filtering independent white noise in the frequency band of interest, in this case we selected the alpha band between 8 and 12 Hz. The power of one time series was manipulated to be negatively correlated to an external variable and projected on the “invariant source”.

Finally, the SNR was calculated as the ratio between the mean variance across channels for the invariant source and the mean variance of additive background noise and the individual oscillatory sources in the centre frequency of the brain source. We generated data with three different signal-to-noise ratios, 0.01, 0.05 and 0.1.

We simulated 45 persons for whom 300 seconds of data were generated at a sampling frequency of 200 Hz. We repeated the analysis for 100 different random positions of the target (invariant) source, to be able to analyse the ability of SPoC to recover the source whose power co-modulates with an external variable and whose orientation is slightly different for each subject.

To assess whether SPoC could correctly recover the pattern of interest, the error between the original and the SPoC pattern was calculated according to [40]:

1 $$\begin{aligned} Err=1-\frac{\vert \varvec{a}_o^T \varvec{a}_{\mathrm{SPoC}}\vert }{\Vert \varvec{a}_o \Vert \Vert \varvec{a}_{\mathrm{SPoC}} \Vert } \end{aligned}$$

where $\varvec{a}_o$ is the original generated pattern and $\varvec{a}_{\mathrm{SPoC}}$ is a pattern recovered by SPoC. The final error estimate was obtained averaging over all errors.

### Real data description

**Fig. 1:**
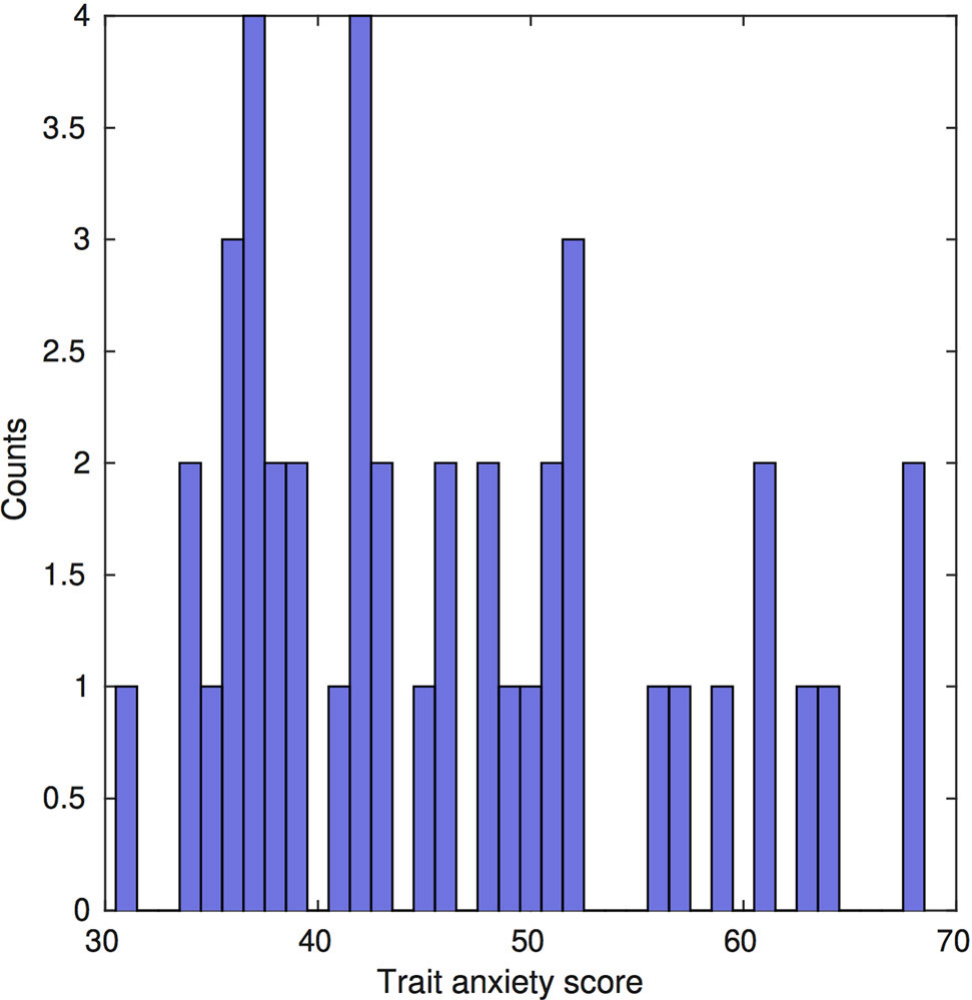
Histogram of the Spielberger trait anxiety scores in our participant sample, with x-axis representing the trait anxiety score of each participant and y-axis the number of participants associated with each score

The data analysed in this paper were obtained from our previous study [41], which was approved by the local ethical review committee at Goldsmiths, University of London. EEG data were available from 43 participants during wakeful rest (5 minutes, eyes open). Participants were on average 27 years old (standard error of the mean or SEM 0.9, range 18–35; 28 females). In this sample, trait anxiety scores were available; these had been obtained with the Spielberger State-Trait scale (STAI, Trait sub-scale, T-STAI, 20 items, score in range 20–80). The trait values in our sample were distributed in the 30–68 range (mean [SEM]: 46 [2]; Fig. 1). T-STAI scores above 45 are considered high, as these values are typically reported in patients with anxiety disorders [42]. Note that in the original study [41], neural and behavioural data during task performance were available in 42 participants, yet resting state recordings were collected for one additional participant (N = 43). In [41], the 42 participants were split into two groups *after* the resting-state recordings. One participant group underwent a manipulation aiming to induce sustained anxiety during task performance (experimental group). The analysis in that study therefore focused on neural and behavioural differences between a control and experimental group. By contrast, the current study focuses on data from all 43 participants prior to the subsequent group manipulations. We refer the reader to [41] for further details.

EEG signals were recorded using the BioSemi ActiveTwo system (64 electrodes, extended international 10–20, sampling rate 512 Hz, high-pass filter 0.1 Hz). External electrodes were placed on the left and right earlobes to use as references upon importing the EEG data in the analysis software. The signals had been pre-processed already as described in [41], using the EEGLAB toolbox [43] for MATLAB^®^. In that study, the continuous EEG data were filtered using a high-pass filter at 0.5 Hz and then notch-filtered at 48–52 Hz. Next, artefacts related to eye blinks, saccades and heartbeats were removed from the signals using independent component analysis (ICA, runICA implementation; 2.3 components were removed on average). See [41] for further details on pre-processing.

**Fig. 2:**
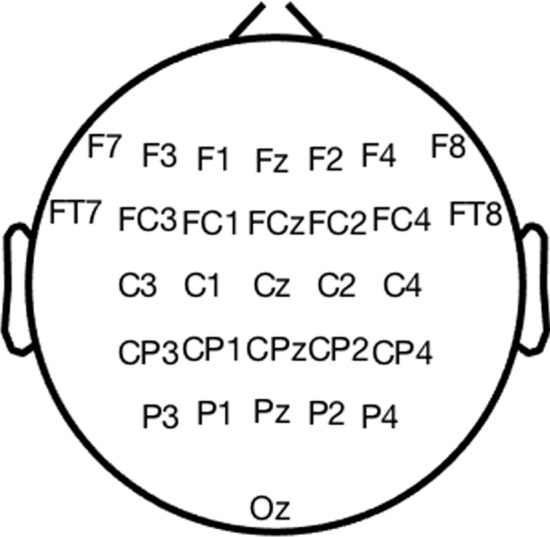
Electrodes selected for the analyses

The analyses performed in this work were based on a subset of 30 electrodes covering the whole scalp (Fig. 2). Artifactual channels were removed by inspecting their variance in the band between 4 and 30 Hz. Those displaying high power were rejected, individually for each participant [44], at the same time trying to maintain as many as possible on the frontal and fronto-central areas [14, 15]). In order to run SPoC over all subjects simultaneously, the set of channels must be common to all data sets. The selected 30 channels were noise free in all participants.

## Methods

### Source power comodulation, SPoC

Source Power Comodulation or SPoC is a method designed to decompose multivariate neuroimaging data (EEG/MEG) into source components by using the information contained in an external target variable to direct the decomposition [35]. As a result, a set of spatial filters is found that optimizes the covariation or correlation (depending on the selected objective function) between the external target z and the power time course of the corresponding SPoC source. That is, SPoC maximizes the correlation between the power of the neural signals and a variable of interest, thereby identifying the filters and patterns maximally related to the variable of interest. Furthermore, the obtained patterns can be neurophysiologically interpreted and their sources located with standard algorithms such as eLORETA [36].

Two different SPoC algorithms exist $\hbox {SPoC}_\lambda$ and $\hbox {SPoC}_{r^2}$. $\hbox {SPoC}_\lambda$ provides an analytical solution and obtaining a result is significantly faster than for $\hbox {SPoC}_{r^2}$, thus it was our preferred option in this work. As described in [35], by maximizing the covariation between a power of a brain source and an external variable, one can arrive at the following optimization problem:

2 $$\begin{aligned} \mathop {\mathrm{arg max}}\limits _{\varvec{w}} { \varvec{w}^T \varvec{C}_z \varvec{w} } \end{aligned}$$

with respect to the following norm constraint:

3 $$\begin{aligned} \varvec{w}^T \varvec{C} \varvec{w} =1 \end{aligned}$$

with $\varvec{C}_z$ being the covariance between the power (in form of a covariance matrix) of the band-pass filtered EEG signal at each epoch and the standardized external value z (with zero mean and unit variance); and $\varvec{C}$ the averaged value of the epoch covariance matrices of the band-pass filtered EEG signal. In the context presented in this manuscript, an epoch is the complete data of one participant. Thus, for *N* participants we would have *N* epochs.

The aforementioned constrained optimization problem can be solved using the method of Lagrange multipliers. Setting the first derivative of the corresponding Lagrangian to zero leads to the following generalized eigenvalue equation:

4 $$\begin{aligned} \varvec{C}_z \varvec{w} = \varvec{\lambda } \varvec{C} \varvec{w} \end{aligned}$$

On the other hand, $\hbox {SPoC}_{r^2}$ directly maximizes the squared correlation between the experimental variable and the power time series:

5 $$\begin{aligned} f_{r^2} = \frac{(\varvec{w}^\top C_z \varvec{w})^2}{\langle (\varvec{w}^\top (\varvec{C} (e)-\varvec{C})\varvec{w})^2\rangle } \end{aligned}$$

with $\varvec{C}(e)$ the covariance matrix of one epoch *e* of data and $\langle \cdot \rangle$ denoting averaging across epochs. According to [35] “The weight vector $\varvec{w}$ that maximizes $f_{r^2}$ cannot be found analytically. It should therefore be found using iterative optimization methods.” This makes $\hbox {SPoC}_{r^2}$ significantly slower than $\hbox {SPoC}_\lambda$.

### Spatio-spectral decomposition, SSD

An important aspect to consider when applying an inter-subject approach of an optimization procedure is that the total number of subjects should largely exceed (typically by a factor of five to ten) the number of unknowns. In SPoC, the number of unknowns is the dimension of the weight vector $\varvec{w}$, which equals the number of selected EEG channels. However, it is common that the number of participants in a study is not much greater or even smaller, than the number of electrodes of interest. One solution to this problem is to apply a dimensionality reduction algorithm.

In this work we selected the spatio-spectral decomposition (SSD) to reduce dimensionality [45–47]. SSD can find spatial filters which maximize the signal-to-noise ratio (SNR) of oscillatory signals, such as band-pass filtered EEG, [48]. This study showed that maximizing the SNR of the measured signals, also maximizes the SNR of the sources of interest. The optimization problem takes advantage of the positive definite property of covariance matrices as power estimators and reduces to a generalized eigenvalue problem.

6 $$\begin{aligned} SNR= & {} \frac{P_s(f)}{P_n(f)} \approx \frac{P_m(f)}{P_m(f-\Delta f) + P_m(f+\Delta f)} \end{aligned}$$

7 $$\begin{aligned} SNR(\varvec{w})= & {} \frac{\varvec{w}^{T} \varvec{\Sigma }_{m} \varvec{w}}{\varvec{w}^{T} \varvec{\Sigma }_{n}\varvec{ w}} \end{aligned}$$

with $P_s(f)$ and $P_n(f)$ the power of the source signal and the noise at a narrow frequency band, respectively, and $P_m(f)$ the power of the measured signal. Finally, $\varvec{\Sigma }_{m}$ and $\varvec{\Sigma }_n$ are the covariance matrices of the measured signal and noise (the later is measured at the flanking frequencies) of the filtered EEG and $\varvec{w}$ is the spatial filter to obtain a source of maximal SNR.

The selection of components was performed by projecting the filtered EEG data into the SSD directions and correlating their power with the external variable. Those five components with higher correlation were selected to then conduct SPoC. The possible over fit was estimated using permutation tests.

### Calculation of sensor-space correlations

The usual way to investigate the association between the power of neuronal oscillations and some behavioural/clinical variable is to directly use channel data. Specifically, sensor-based correlations were computed with respect to a variable of interest in simulated and real EEG data as described in the next sections.

#### Simulated EEG data

We computed 30 small-Laplacian derivations from the original 30 channels by allowing computations with an incomplete number of neighbouring channels. They were filtered in the alpha band (8 to 12 Hz) and the variance of each channel and each person (trial) was computed. Then, the correlation of the trial-wise variance and the external variable was calculated. Finally, the most significant result was selected. We employed the Spearman correlation coefficient which is robust against the presence of outliers.

#### Real EEG data

The sensor signals of the resting-state recording were spatially filtered using small-Laplacian derivations to obtain 30 Laplacian channels by allowing computations with an incomplete number of neighbouring channels. Then, the data were filtered in three narrow bands corresponding to theta (4–7 Hz), alpha (8–12 Hz) and beta ranges (13–30 Hz). Subsequently, the data were cropped into 2 second windows.

For each of those windows, the variance of a sensor was computed and averaged across windows, obtaining one power value per sensor. Finally, the correlation of each sensor to the external value (trait anxiety score) was computed. We estimated both Spearman and Pearson correlations. Note that power is a measure that often presents outliers. A way to reduce their influence is to apply a logarithm to power values before computing correlations, thus all Pearson correlations were performed with log-power values.

### Application of inter-subject SPoC to simulated and real data

#### Simulated EEG data

SSD was applied to the simulated EEG data in the band 8–12 Hz. Then, five SSD directions were selected as described in Sect. 3.2. After that SPoC was computed to those five components and the correlation between the power of the selected SPoC component and the external variable calculated. Again, we employed Spearman correlation due to its robustness against possible outliers.

Finally, in order to show the advantage of applying SSD previous to SPoC, we also computed SPoC directly from the band-pass filtered sensor data.

#### Real data

The EEG data were pre-processed as in Sect. 3.3, i.e. they were filtered in narrow bands and cropped into 2-second long windows. Then, instead of the variance of each electrode, the corresponding covariance matrix was computed for each window and averaged over epochs. In order to apply SSD, also noise covariances were computed following the same procedure, with the exception that the data were filtered in the flanking frequency bands. Those signal and noise covariance matrices were used to compute SSD and five components were selected as in Sect. 3.2. The EEG data were then projected into those components and then both SPoC algorithms together with the standardized trait score results of each participant were applied. To analyse the results we computed both Spearman and Pearson correlations, in this last case using log-power values.

Spatial patterns in sensor space could be recovered by multiplying the resulting SPoC pattern in SSD space with the matrix formed with the five selected SSD patterns. Then, this sensor-space pattern could be located using eLORETA [49].

### Statistical analyses

#### Significance of the results

The confidence limit for the correlation obtained with SPoC in real and simulated data was estimated with permutation tests [50]. One thousand permutations were performed and for each of them, where all optimization steps were repeated using a shuffled external variable. After computing SSD, the five components were selected by correlating the shuffled variable with the power of the projected data. Then, SPoC was applied and the Spearman correlation of the selected SPoC component and the shuffled variable was estimated. In the case of real data, the results with Pearson correlation between the log-power and the permuted trait anxiety index were also computed. Positive significant correlation values of the original data were those exceeding the 97.5 percentile of permuted correlations. In case of negative correlations, significant results were those below the 2.5 percentile of permuted correlations. Furthermore, the *p* values were estimated as the proportion of permuted correlation values which exceeded (for positive correlations) or were smaller (for negative correlations) than the original unpermuted result.

#### Differences between methods

Differences between methods were assessed using Friedman tests because correlation results were not normally distributed [51]. In case the result was significant, post hoc analyses were performed using the Nemenyi test, an equivalent to Tuckey’s HSD for nonparametric testing [52]. Only factor “Method” was considered for these tests. SNR was not included as a factor because it is clear that higher SNRs produce better results. Furthermore, SNR is not known for real data.

## Results

### Simulations

Averaged correlation results over 100 repetitions obtained by simulations are summarized in Table 1. There, it is visible that for higher SNRs (0.1 and 0.05), all SPoC studied methods (both SPoC variants with and without SSD) deliver very similar outcomes. However, for SNR=0.01 $\hbox {SPoC}_{r^2}$ seems to strongly overfit (obtains on average positive correlations) in comparison to the rest of SPoC methods. Differences between methods were studied using Friedman tests, one for each SNR, with all results being significant (*p* value$<< 0.001$). Thus, post hoc tests followed. In the case of SNR=0.1 and SNR=0.05, the differences between SPoC-related methods were not found to be significant, whereas the best Laplacian derivation obtained significantly worse results than all SPoC results. In the case of SNR=0.01, $\hbox {SPoC}_{r^2}$ was found significantly worse than the rest of SPoC methods and also worse than the best Laplacian derivation. Otherwise the results with the best Laplacian derivation were significantly worse than using all other SPoC-related methods.

**Table 1:** Averaged Spearman correlation values over 100 repetitions ± standard error of the mean

SNR	$\hbox {SPoC}_\lambda$	SSD+$\hbox {SPoC}_\lambda$	$\hbox {SPoC}_{r^2}$	SSD+$\hbox {SPoC}_{r^2}$	Best Lap.
0.1	− 0.960 ± 0.003	− 0.961 ± .003	− 0.960 ± 0.003	− 0.961 ± 0.003	− 0.845 ± 0.016
0.05	− 0.908 ± 0.007	− 0.914 ± 0.005	− 0.739 ± 0.055	− 0.915 ± 0.005	− 0.741 ± 0.020
0.01	− 0.554 ± 0.019	− 0.602 ± 0.016	0.236 ± 0.073	− 0.611 ± 0.016	− 0.421 ± 0.016

Averaged pattern recovery errors over 100 repetitions are presented in Table 2. Note that Laplacian derivations do not return patterns, thus a recovery error cannot be computed. Again, SPoC variants with SSD seem to perform better than those without dimensionality reduction when the SNR is low. Friedman tests with factor “Method” returned significant results for all SNRs. Differences were not found significant between SPoC methods without dimensionality reduction for any of the SNRs. On the other hand $\hbox {SPoC}_\lambda$ was significantly better than the rest of methods for all SNRs.

**Table 2:** Averaged source recovery errors over 100 repetitions ± standard error of the mean

SNR	$\hbox {SPoC}_\lambda$	SSD+$\hbox {SPoC}_\lambda$	$\hbox {SPoC}_{r^2}$	SSD+$\hbox {SPoC}_{r^2}$
0.1	0.00061 ± 0.00004	0.00031 ± 0.00003	0.00061 ± 0.00004	0.00210 ± 0.00087
0.05	0.00192 ± 0.00034	0.00069 ± 0.00008	0.07020 ± 0.020901	0.00465 ± 0.001495
0.01	0.31175 ± 0.03396	0.08981 ± 0.01992	0.49903 ± 0.03330	0.09426 ± 0.01702

**Fig. 3:**
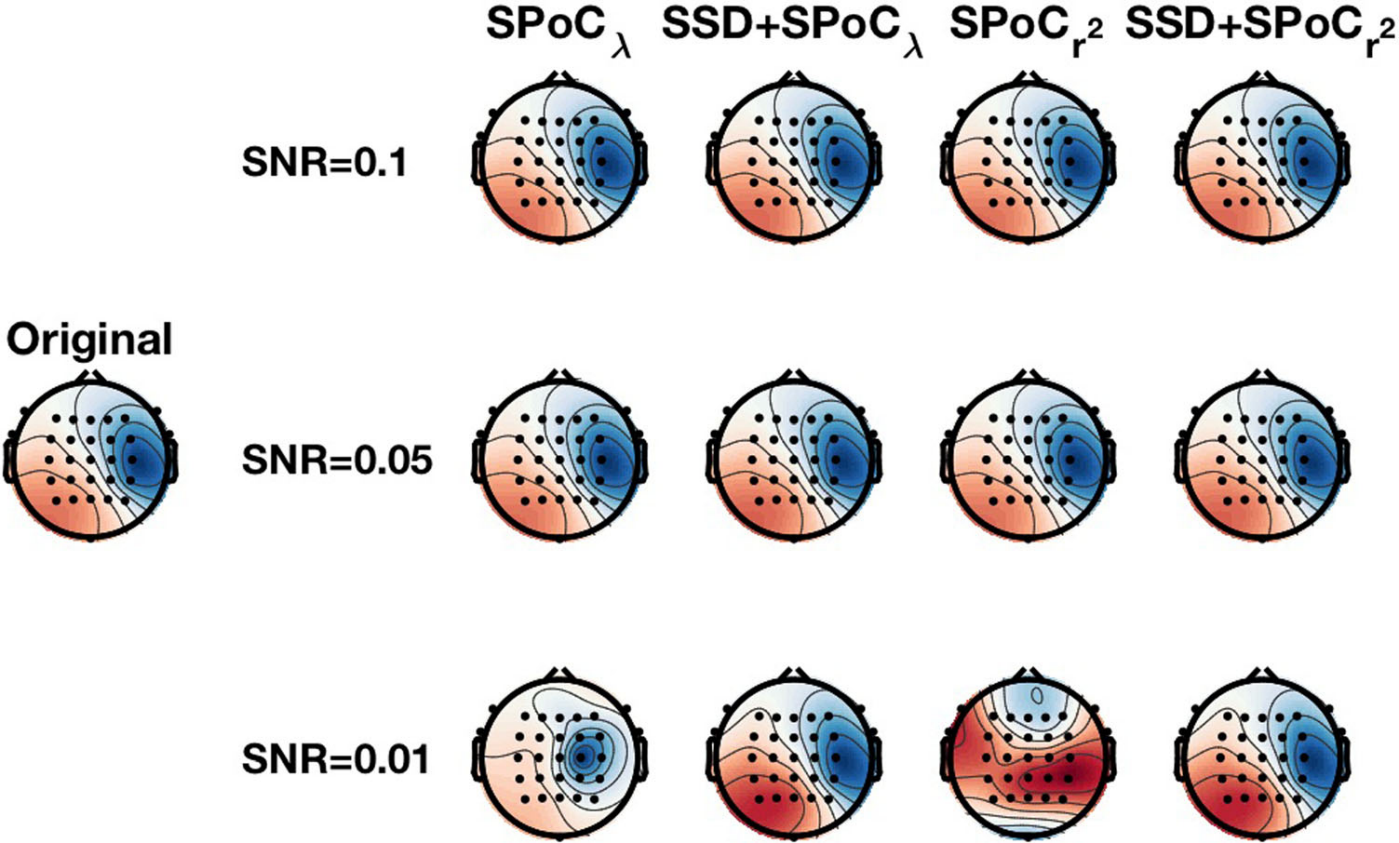
Left: Original generated pattern. Rest: recovered patterns of SPoC algorithms with and without SSD for the three SNR studied

Finally, an example of a recovered pattern is visible in Fig. 3. The first column displays the original pattern, whereas the rest display results with different SPoC versions. Each row presents one SNR with 0.1 on the top. For the lowest SNR, SPoC versions with SSD find the correct pattern, whereas without SSD the result does not resemble the original pattern, specially for $\hbox {SPoC}_{r^2}$ with the highest recovery error.

### Real data

We applied both SPoC algorithms to three different frequency bands, namely theta, alpha and beta. Real data are usually more noisy than simulated data, thus we employed SSD and selected five components prior to the application of SPoC. Next, SPoC extracted the source patterns that had power maximally correlated (positively and negatively) with the external variable (trait anxiety score of each participant). We used permutation tests to assess the statistical significance of the results. We also employed both Spearman and Pearson correlation coefficients to assess the results. However, as Pearson can be heavily affected by data outliers, we applied a logarithm to the extracted power values to improve the result.

**Fig. 4:**
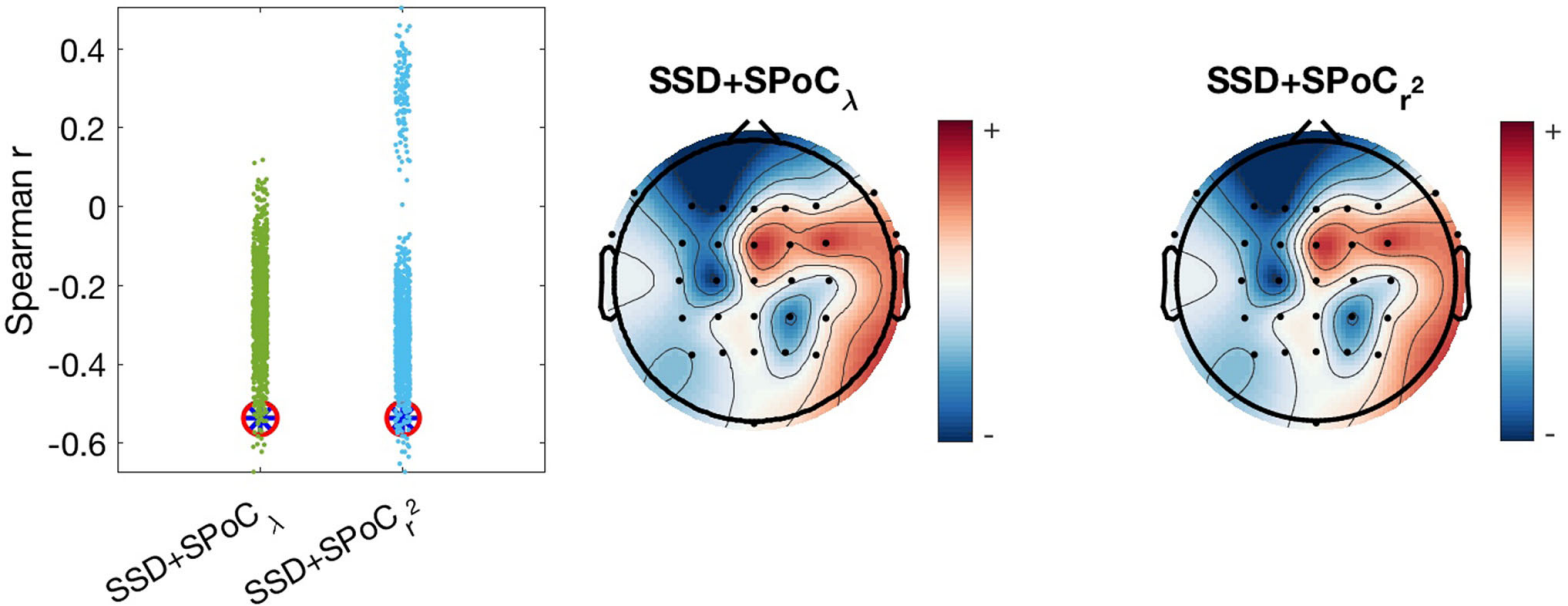
Left: Spearman correlation obtained for SSD+$\hbox {SPoC}_\lambda$ and SSD+$\hbox {SPoC}_{r^2}$ (circled in red) and for each permutation (points in green and blue, respectively). To achieve these results, the external variable was shuffled previously to the selection of SSD components and the corresponding SPoC variant applied. After that, power features were extracted and the Spearman correlation coefficient obtained with the shuffled variable. Right: SSD+SPoC pattern of sources for the results shown on the left

The results obtained with both SPoC algorithms and both correlation options are presented in Table 3. As expected, the outcomes of either method were similar. In particular, power in the theta band is significantly anti-correlated with the trait anxiety score, with a very similar value for Spearman and Pearson methods (− 0.54, − 0.53, respectively; see Table 3 and Fig. 4). The corresponding *p* values from the permutation tests are also shown in Table 3, (0.016 and 0.028 for Spearman and 0.016 and 0.018 for Pearson, respectively). These outcomes indicate that larger power values in the obtained spatial pattern are associated with lower trait anxiety across participants. No other significant effects were found for alpha and beta bands.

**Table 3:** Correlation results and p-values for each of the SPoC algorithms and each of the studied bands. Results in bold face are significant

Band		Correlation results		*p* values	
		SSD+$\hbox {SPoC}_\lambda$	SSD+$\hbox {SPoC}_{r^2}$	SSD+$\hbox {SPoC}_\lambda$	SSD+$\hbox {SPoC}_{r^2}$
**Theta**	Spearman	$-$ **0**.**54**	$-$ **0**.**54**	**0**.**016**	**0**.**028**
	Pearson	$-$ **0**.**53**	$-$ **0**.**53**	**0**.**016**	**0**.**018**
Alpha	Spearman	−0.34	−0.34	0.314	0.403
	Pearson	−0.38	−0.38	0.227	0.297
Beta	Spearman	−0.30	−0.30	0.417	0.552
	Pearson	−0.34	−0.34	0.346	0.461

**Fig. 5:**
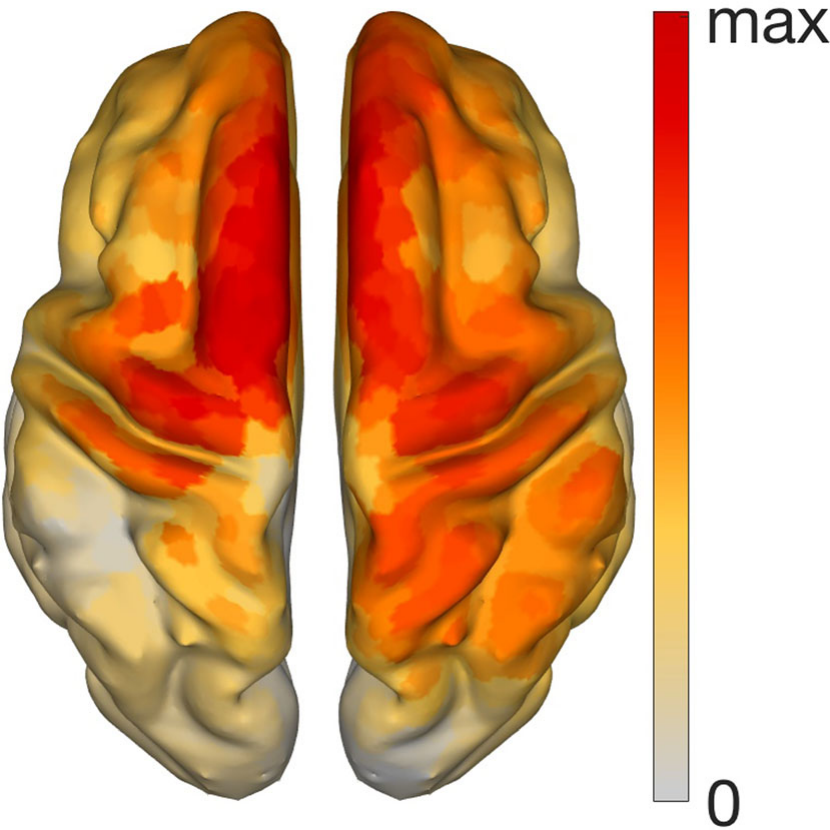
eLORETA localization of the significant SSD+$\hbox {SPoC}_\lambda$ pattern at theta band. The resulting SSD+$\hbox {SPoC}_{r^2}$ is the same

The corresponding patterns obtained with SSD+$\hbox {SPoC}_\lambda$ and SSD+$\hbox {SPoC}_{r^2}$ are displayed in the middle and right panels of Fig. 4. These topographies correspond to the strongest association between power of theta oscillations and the anxiety score. Both patterns are almost equal, and display a difference of only $2.3 \, 10^{-16}$, supporting that both SPoC algorithms return the same pattern in these data. The strongest activity is observed over centro-frontal areas. Correspondingly for this pattern, inverse modelling using eLORETA revealed neuronal sources being located bilaterally in the superior frontal gyrus extending posteriorly to the precentral gyrus (Fig. 5).

**Fig. 6:**
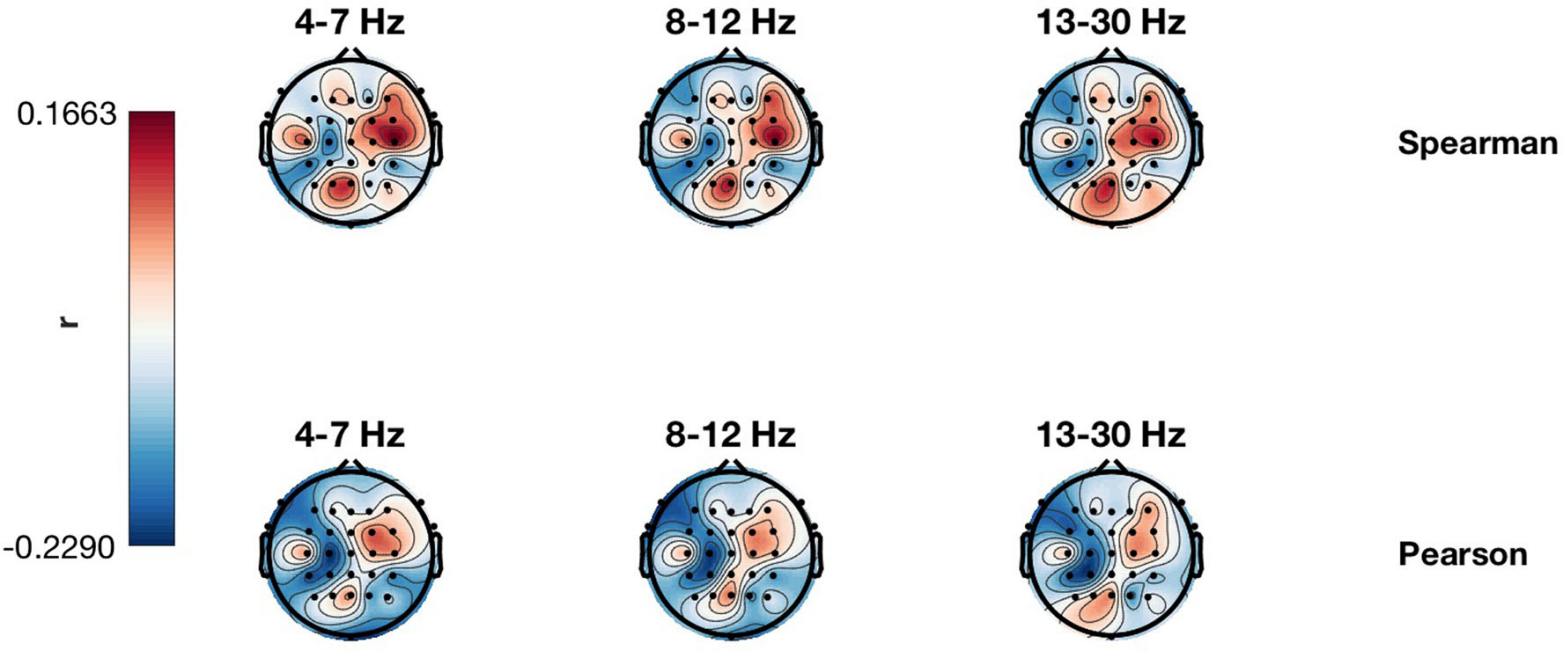
Distribution over the scalp of the correlation results for each frequency band (theta, 4–7 Hz; alpha, 8–12 Hz; beta, 13–30 Hz). Red denotes positive correlation values, while blue represents negative correlations. None of these results were significant

Complementing the SSD+SPoC analyses, we also obtained Spearman and Pearson correlation results in sensor-space for the same frequency bands (4–7, 8–12 and 13–30 Hz). Among all Laplacian channels, we selected those with the most extreme correlation values for each band. The results are shown in Table 4. None of the results was significant in any of the channels or bands, although some results showed a trend towards significance. The distribution over the scalp of non-significant correlation results is shown in Fig. 6.

**Table 4:** Correlation results and p-values for the best Laplacian channel and each of the studied bands. None of the results are significant

Band	Correlation results		*p* values	
	Spearman	Pearson	Spearman	Pearson
Theta	− 0.194	− 0.229	0.106	0.070
Alpha	− 0.192	− 0.229	0.108	0.075
Beta	− 0.205	− 0.214	0.093	0.084

The distributions of correlation values in the scalp using Laplacian channels seem to be very similar in all studied bands and for both correlation coefficients. However, none of the results is significant. Furthermore, as aforementioned, the obtained spatial distributions from Laplacian channels cannot be interpreted in terms of sources because the activity in each channels is first squared (to obtain power) and only then correlated with the anxiety. Therefore, the polarity of sources is lost and thus no meaningful neurophysiological interpretation is possible. As a consequence inverse modelling, such as eLORETA cannot be applied to these results.

## Discussion

The present work aims to identify neurophysiological markers in subclinical trait anxiety that could be used as intervention targets for neurofeedback and neuromodulation studies. Unlike other EEG paradigms, where discrete classes are identified and discriminated [53–59], here we deal with continuous scores or levels of anxiety obtained from T-STAI. The main finding is the extraction of a spatial pattern in the theta band of resting state oscillatory EEG activity. This pattern is significantly and maximally associated with trait anxiety across participants. Furthermore, it can also be directly interpreted as a source pattern, and is localised primarily to the superior frontal gyrus using eLORETA. The results across simulated and real EEG data validate the usefulness of SPoC as a method for identification of markers of neurophysiological activity optimally representing psychological or behavioural features.

Previous work typically conducted standard correlation analyses between sensor-level EEG activity and an external variable of interest to establish associations between behavioural or psychological features and neural data [35]. This approach has limitations, however, as the mixing of source and noise activity at the scalp renders those results non-interpretable. In particular, when the computation of the neural measures involves nonlinear operations, establishing an association between the individual electrode estimates and their sources in the brain is not possible anymore. This is the case with regard to spectral power estimates, which rely on squaring the frequency-transformed data. Moreover, this operation removes phase information in the data, which is important for the adequate reconstruction of neuronal sources. Accordingly, classical sensor-level correlation results on the scalp cannot be related to the neuronal origin of the observed effects.

Our simulation results show that both SPoC variants can recover the true inter-subject source whose power is correlated with an external variable. The addition of SSD as a pre-processing step turns both methods robust against overfit (see Table 1), returning the sources of interest more efficiently (Tables 2 and 3). Such advantage of SSD to avoid overfitting has been also previously shown earlier [45, 46]. In particular, both SSD+SPoC algorithms are able to recover the artificially generated pattern of sources in all investigated conditions, whereas this was not the case for any SPoC alone method (see Table 2 and Fig. 3 for an exemplary result). Notably, according to our statistical analyses, SSD+$\hbox {SPoC}_\lambda$ is the most reliable method to recover sources. The results also demonstrate that SSD+SPoC-related correlations are stronger than those based on sensor-space results. Furthermore, $\hbox {SPoC}_r{^2}$ strongly overfits for the lowest SNR studied, in comparison also to $\hbox {SPoC}_\lambda$. These outcomes suggest that $\hbox {SPoC}_\lambda$, apart from being faster to calculate is also more robust against noise. Also, our simulations show that inter-subject SPoC can be a useful procedure to obtain neurophysiological markers optimised for an external target variable, in addition to being interpretable as neural sources and, thus, informative for neuroscientific research.

With respect to the analysis of empirical EEG data, SSD+SPoC ($\lambda$ and $r^2$) reveals a spatial pattern with its corresponding activity being maximally comodulated with trait anxiety. This was specific to the theta band; SSD+SPoC values in the alpha and beta bands were non-significant in any of the studied cases (both SPoC methods and both correlation coefficients, Spearman and Pearson). Moreover, the associated nonparametric correlation values for the theta band are stronger (− 0.54) than those observed in the standard analysis of sensor-space correlations (− 0.194 for Spearman − 0.229 for Pearson in theta band, and similarly for alpha and beta).

Beyond SPoC, the use of spatial filters aiming to optimise a target measure is widespread in BCI applications [46]. Common spatial patterns, for instance, which maximise the variance of the spatially filtered signal in one condition, while minimising it in a second condition, have been shown to increase online BCI decoding accuracy [60–63]. Here, using SSD+SPoC, we identify a theta-band spatial pattern that could be a candidate target in resting-state EEG neurofeedback studies, potentially increasing the efficacy of the neurofeedback-based modulation of neural activity and associated level (scores) of trait anxiety. This is a promising extension of previous neurofeedback work in anxiety, which primarily used theta or alpha oscillatory power [3].

The choice of theta and alpha power in previous research was based on the vast evidence linking theta with deep relaxation states, and decreased arousal, similarly to alpha activity [64, 65]. In addition to managing arousal and facilitating relaxation, alpha-based neurofeedback training has been shown to decrease trait anxiety levels, and increase the feeling of control in stressful settings [28, 66]. While theta oscillations have been more widely used as target for neurofeedback interventions in post-traumatic stress disorder than in anxiety [3], our findings suggest a specific spatial pattern (and a corresponding spatial filter) that could be used in BCI protocols to test the efficacy of theta-band neurofeedback for anxiety. The spatial pattern findings are complemented with the source analysis using eLORETA, which localised the theta-band spatial pattern in the superior frontal gyrus. This region is associated with frontal midline theta in anxiety, in addition to cognitive control [26]. Crucially, a large body of neuroimaging evidence demonstrated that regions of the prefrontal cortex and anterior cingulate cortex, which are part of the cognitive control network, overlap with the circuitry of anxiety [67, 68]. Along a similar line, the superior frontal gyrus has been associated with anxiety, in particular with social anxiety disorders [69], while it is also involved in cognitive control [70].

## Conclusion

Our study presents a novel framework for the optimal identification of neural cortical biomarkers relating to anxiety. It aims at the identification of neural sources that are invariant across subjects. Our results are encouraging and can be potentially used to monitor neuronal activity as a result of therapeutic manipulations to alleviate anxiety. Our framework uncovered a biomarker from resting-state EEG that is in line with previous literature, yet future work using a larger number of participants will be necessary to refine the spatial patterns/filters observed here.

## Data Availability

The datasets generated during and/or analysed during the current study are available from the corresponding author on reasonable request.
